# Editorial: Insights in coronary artery disease: 2023

**DOI:** 10.3389/fcvm.2025.1619625

**Published:** 2025-05-15

**Authors:** Giuseppe Ando', Felice Gragnano, Marco Giuseppe Del Buono, Viktor Kočka, Marco Zimarino, Toshiro Shinke, Krishnaraj Rathod, Javed Ahmed, Alberto Guido Pozzoli, Tommaso Gori

**Affiliations:** ^1^ Department of Clinical and Experimental Medicine, University of Messina, Messina, Italy; ^2^ Department of Translational Medical Sciences, University of Campania “Luigi Vanvitelli”, Caserta, Italy; ^3^ Department of Cardiology, Agostino Gemelli University Polyclinic (IRCCS), Rome, Italy; ^4^ Department of Cardiology, University Hospital Kralovske Vinohrady and Third Faculty of Medicine, Charles University, Prague, Czechia; ^5^ Department of Cardiology, ASL Lanciano Vasto Chieti, Chieti, Italy; ^6^ Division of Cardiology, Department of Medicine, Showa University School of Medicine, Tokyo, Japan; ^7^ Centre for Cardiovascular Medicine and Devices, Queen Mary University of London, London, United Kingdom; ^8^ Department of Cardiology, Newcastle upon Tyne Hospitals NHS Foundation Trust, Newcastle upon Tyne, United Kingdom; ^9^ Istituto Cardiocentro Ticino, Ospedale Regionale di Lugano, Lugano, Switzerland; ^10^ Zentrum für Kardiologie, University Medical Centre, Johannes Gutenberg University Mainz, Mainz, Germany

**Keywords:** coronary artery disease, atherosclerosis, myocardial infarction, percutaneous coronar intervention, inflammation

**Editorial on the Research Topic**
Insights in coronary artery disease: 2023

Coronary artery disease (CAD) continues to pose a substantial global health burden, remaining one of the leading causes of death and disability across both developed and developing nations. Despite decades of progress in risk factor management (1), revascularization strategies (2), pharmacological treatments (3), and imaging technologies (4), significant challenges persist in achieving optimal prevention, diagnosis, and long-term outcomes. The Research Topic *“Insights in Coronary Artery Disease: 2023”*, hosted by *Frontiers in Cardiovascular Medicine*, presents a curated Research Topic of original research articles, reviews, and case reports that offer cutting-edge perspectives on various aspects of CAD—from interventional cardiology and intravascular imaging to digital modeling, inflammation, and even the gut-heart axis.

One of the central themes emerging from this Topic is the pursuit of better risk prediction and personalized care pathways for patients with CAD. Dai et al. developed and validated a user-friendly nomogram to predict the five-year risk of non-culprit lesion (NCL) revascularization in patients with ST-segment elevation myocardial infarction (STEMI) who had undergone primary percutaneous coronary intervention (PCI). Their model integrates clinical and laboratory variables such as age, body mass index, Killip class, and low-density lipoprotein cholesterol (LDL-C) levels to identify individuals at elevated long-term risk. The clinical relevance of this tool lies in its potential to inform more individualized surveillance, medical therapy intensification, and follow-up strategies tailored to residual risk—particularly important in the era of complete revascularization. Notably, prior studies (5) have also emphasized the prognostic value of non-culprit lesion assessment following STEMI, underscoring the need for refined risk stratification approaches.

Sex differences in outcomes following complex coronary interventions remain an area of growing interest (6). In a robust analysis from a nationwide Spanish registry, Alperi et al. demonstrated that women undergoing complex PCI had a significantly higher risk of all-cause mortality and myocardial infarction compared to men. This disparity persisted despite adjustment for comorbidities and procedural complexity. Their findings underscore the need for continued investigation into sex-specific pathophysiology, differences in response to therapy, and underrepresentation of women in clinical trials—factors that may ultimately influence guideline recommendations and improve outcomes for female patients.

Technological advancements in stent platforms also feature prominently in this collection. The CAESAR registry, reported by Tarantini et al., evaluated the real-world performance of a novel ultrathin-strut, polymer-free sirolimus-eluting stent (Coroflex ISAR NEO) in an all-comers population with CAD. The results were encouraging, with low rates of target lesion revascularization and major adverse cardiac events at one year, confirming the safety and efficacy of this new-generation stent. These findings contribute to the growing body of evidence supporting polymer-free platforms, which may reduce chronic inflammation and improve endothelial healing.

Understanding the mechanistic basis of atherosclerosis progression and plaque rupture is key to preventing acute coronary syndromes. Bacigalupi et al. provided a comprehensive review on the role of biomechanical forces—particularly wall shear stress, axial plaque stress, and structural stress—in shaping plaque vulnerability. With the advent of high-resolution imaging and computational fluid dynamics (CFD), it is now possible to model these forces in patient-specific coronary geometries. This approach holds promise for identifying plaques at high risk of rupture before clinical events occur, thus enabling preemptive interventions.

In line with this, Lashgari et al. proposed the use of in silico 3D modeling as a novel adjunctive tool in the cardiac catheterization laboratory. They demonstrated how patient-specific coronary models, generated from angiographic data and processed using CFD, can provide functional insights beyond visual assessment. Such models may enhance diagnostic precision, especially for intermediate lesions where functional significance is uncertain. As digital health tools evolve, the integration of modeling and simulation into clinical workflows could revolutionize decision-making in interventional cardiology.

The inflammatory component of atherosclerosis remains a focal point for research, particularly as imaging tools allow for more nuanced plaque characterization. Los et al. reviewed current invasive coronary imaging techniques—such as intravascular ultrasound (IVUS), optical coherence tomography (OCT), and near-infrared spectroscopy (NIRS)—that can detect plaque features associated with inflammation and vulnerability. These modalities offer valuable prognostic information and may soon be integrated into risk algorithms to guide therapy beyond angiographic stenosis alone. Recent studies (7) have further highlighted the potential of imaging-derived inflammatory biomarkers to refine cardiovascular risk assessment and personalize therapeutic strategies.

The systemic nature of CAD, particularly in elderly patients, was addressed in a study by Xu et al., who evaluated the association between the Geriatric Nutritional Risk Index (GNRI) and the presence of sarcopenia, osteoporosis, and cognitive impairment in older adults with coronary disease. Their findings reinforce the importance of assessing frailty and nutritional status as part of a comprehensive management plan. As the population ages, addressing these geriatric syndromes in CAD patients is increasingly vital to improving quality of life and reducing rehospitalizations.

In an innovative study exploring the gut-heart axis, Hu et al. employed Mendelian randomization to investigate potential causal links between gut microbiota composition and coronary heart disease. Their analysis suggested that the presence of certain microbial genera—including *Bifidobacterium* and *Butyricicoccus*—may have protective or deleterious effects on CAD risk. While still exploratory, this line of research opens the door to microbiota-targeted preventive strategies and underscores the systemic and immunological dimensions of atherosclerosis.

Finally, Dall'Ara et al. presented a compelling case report of recurrent Takotsubo syndrome triggered by asymptomatic SARS-CoV-2 infection, adding to the evolving understanding of COVID-19's cardiovascular sequelae. The case highlights how viral infections, even in the absence of respiratory symptoms, can precipitate acute cardiac syndromes via neurohormonal (8) or inflammatory pathways. It also reinforces the importance of vigilance and multidisciplinary care in the post-COVID era.

In conclusion, the *Insights in Coronary Artery Disease: 2023* Research Topic underscores the significant breadth and depth of contemporary research in coronary artery disease, encompassing advancements in predictive modeling, stent technologies, digital innovation, and the understanding of biological, mechanical, and systemic contributors to disease progression.

The contributions assembled herein highlight the critical importance of interdisciplinary collaboration and the ongoing shift toward precision cardiovascular medicine. Collectively, these studies provide both a comprehensive resource and a strategic framework to guide future investigations, with the ultimate aim of improving clinical outcomes for patients with coronary artery disease.

It is our aspiration that this collection will serve as a valuable reference and a source of inspiration for researchers, clinicians, and trainees committed to advancing the field.
